# Is Indocyanine Green the New Gold Standard for Checking Completion of Laparoscopic Heller’s Cardiomyotomy?

**DOI:** 10.7759/cureus.75344

**Published:** 2024-12-08

**Authors:** Jainil Patel, Vishakha Kalikar, Roysuneel Patankar, Avinash Supe

**Affiliations:** 1 Department of Surgery, Zen Hospital, Mumbai, IND

**Keywords:** achalasia cardia, dor fundoplication, indocyanine green (icg), intraoperative icg, laparoscopic heller's cardiomyotomy, oesophagogastroduodenoscopy (ogd)

## Abstract

Achalasia cardia is a primary motility disorder of the esophagus marked by the absence of peristalsis and the failure of the lower esophageal sphincter (LES) to relax during swallowing. The preferred surgical approach is laparoscopic Heller’s cardiomyotomy with Dor’s fundoplication. Given the significant risks of mucosal perforation and the possibility of incomplete myotomy, which can lead to symptom recurrence, it is essential to ensure both the completeness of the myotomy and the preservation of the mucosal integrity. In this study, we present a case series of 15 patients diagnosed with achalasia cardia who underwent laparoscopic Heller’s cardiomyotomy with Dor’s fundoplication. Intraoperatively, we utilized intraluminal administration of indocyanine green (ICG) dye as an alternative to endoscopy to assess the completeness of the myotomy and to check for any mucosal perforations.

## Introduction

Achalasia cardia is a rare primary motility disorder of the esophagus with a reported annual incidence of 1 to 3 per 100,000 in the Western world. It is characterized by the absence of esophageal peristalsis and incomplete relaxation of a frequently hypertensive lower esophageal sphincter (LES) in response to swallowing [1].

The pathological changes associated with achalasia primarily consist of myenteric inflammation, injury to and subsequent loss of ganglion cells, and fibrosis of myenteric nerves [2]. Although a definitive trigger has yet to be identified, the likely pathogenesis is believed to be autoimmune-mediated destruction of inhibitory neurons in genetically susceptible individuals in response to an unidentified stimulus [3].

The current treatment modalities for achalasia cardia include nonsurgical options such as oral pharmacologic therapy, endoscopic pharmacologic therapy such as Botox injections, and pneumatic dilatation. The surgical options include laparoscopic Heller’s cardiomyotomy and endoscopic options like peroral endoscopic myotomy (POEM) [4].

With a 12.6% incidence of intraoperative esophageal mucosal perforations [5] and the possibility of incomplete myotomy resulting in the recurrence of symptoms, it becomes critical to confirm the completeness of myotomy and mucosal integrity at the end of laparoscopic Heller’s cardiomyotomy. Current confirmatory methods include intraoperative oesophagogastroduodenoscopy (OGD scopy) and dynamic air leak testing. Alternatives such as esophageal manometry, methylene blue dye studies, and the recently introduced use of near-infrared fluorescence using indocyanine green (ICG) dye may be considered.

We present a case series of 15 patients with achalasia cardia undergoing laparoscopic Heller’s cardiomyotomy with Dor’s fundoplication. Intraluminal administration of ICG dye was used intraoperatively as an alternative to endoscopy to evaluate the completeness of the myotomy and to check for mucosal perforations.

The primary objective of this study is to evaluate the utility of ICG in checking the completeness of Heller's cardiomyotomy and the identification of mucosal integrity.

## Materials and methods

The following study is a case series conducted at a tertiary care center in Mumbai for a duration of 24 months from March 2022 to March 2024. This is an extension of our pilot study [6] and has patient overlap. A total of 15 patients with achalasia cardia diagnosed on esophageal manometry were included in the study (8 male patients and 7 female patients), out of which 8 patients had type 1 achalasia cardia, 6 patients had type 2 achalasia cardia, and 1 patient had type 3 achalasia cardia. This study included patients aged 18-60 years, patients fit for general anesthesia, and patients diagnosed with achalasia cardia on OGD scopy and esophageal manometry, including type 1, type 2, and type 3. We excluded patients who were unfit for general anesthesia and those who were older than 18 or older than 60. IRB/IEC approval was deemed inapplicable for this study. Informed consent through the opt-out method was taken. All patients underwent Heller’s cardiomyotomy with Dor's fundoplication, and ICG dye was used intraoperatively to confirm the completeness of the myotomy.

Surgical technique

Preoperatively, all patients were kept on a diet of clear liquids for 24 hours, and a nasogastric tube was inserted. Patients were in a split-leg position with the surgeon standing between the legs and the monitor positioned towards the patient’s left shoulder.

**Figure 1:**
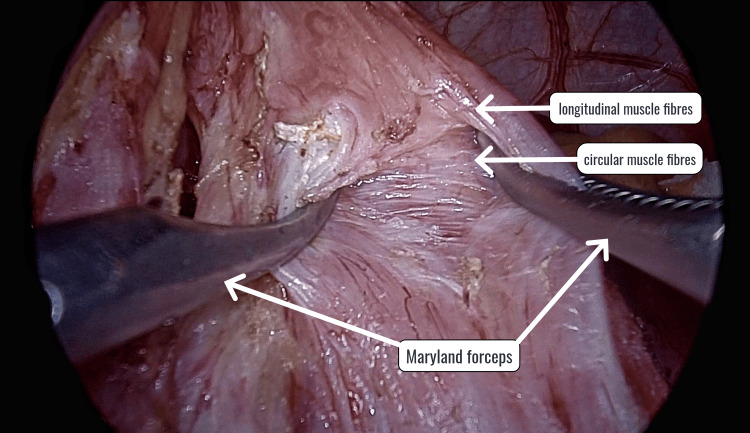
Separation of longitudinal and circular muscle fibers using two Maryland forceps

The pars flaccida was opened, and the phreno-esophageal ligament was dissected to identify the right crus. The anterior vagus was identified as it crossed from left to right at the gastroesophageal junction and was preserved. The Belsey’s pad of fat was dissected off the gastroesophageal junction. Using two Maryland forceps and blunt traction, the longitudinal and circular muscle fibers of the esophagus were separated 5 cm above the esophagus and 3 cm onto the stomach side (Figure 1).

**Figure 2:**
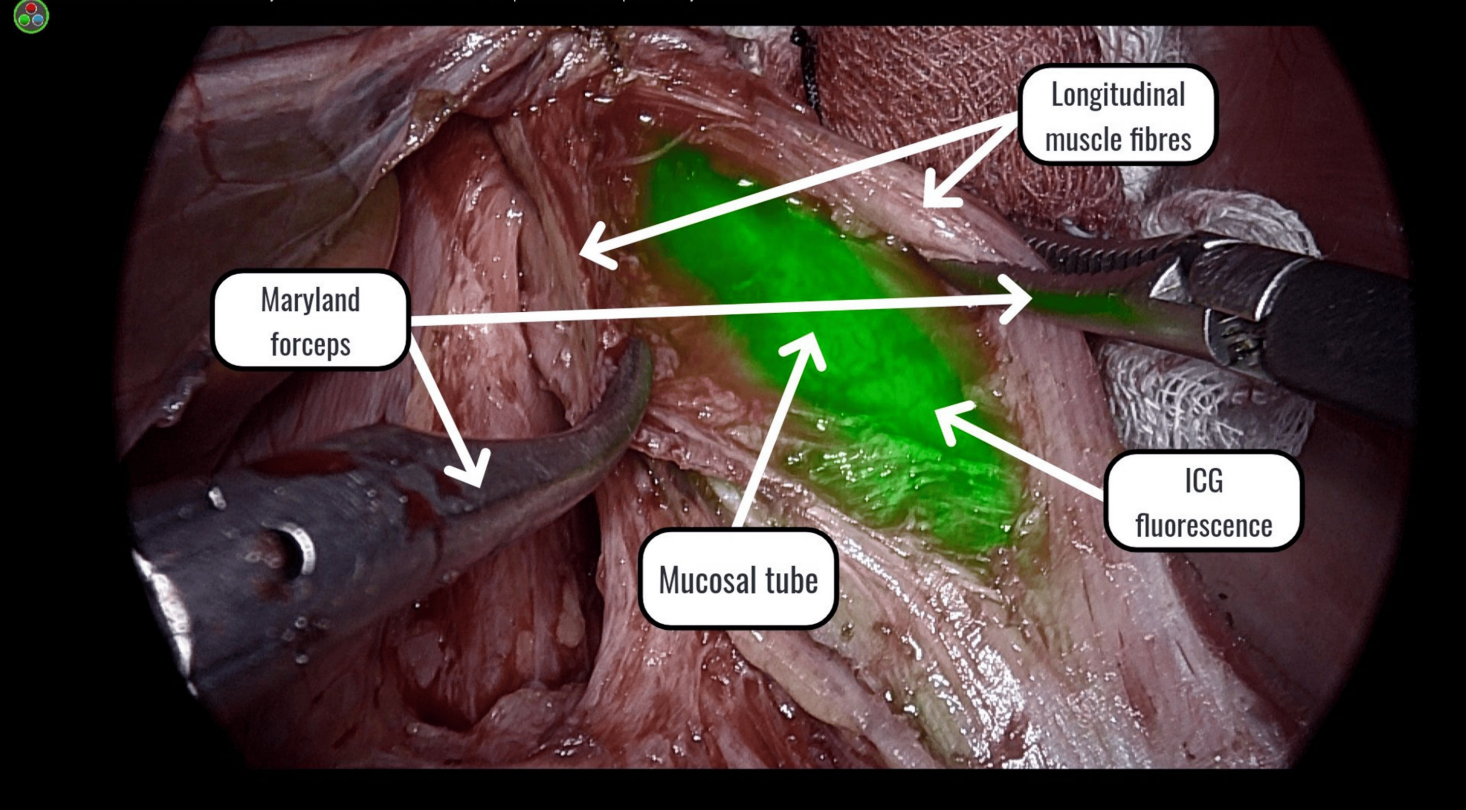
ICG fluorescence ICG: indocyanine green dye

Separation was continued until the long mucosal tube was visualized. The nasogastric tube was then withdrawn to 20 cm, and ICG (10 mg in 100 ml NS) was injected through the tube in the esophagus. The camera was switched to near-infrared fluorescence mode, and ICG was visualized across the mucosal tube immediately after injection (Figure 2).

**Figure 3:**
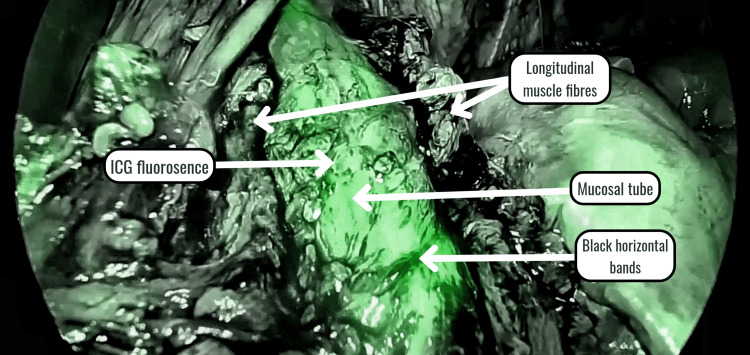
Black horizontal bands representing residual muscle fibers

Residual muscle fibers were visualized as horizontal black bands using the ICG mode (Figure 3).

**Figure 4:**
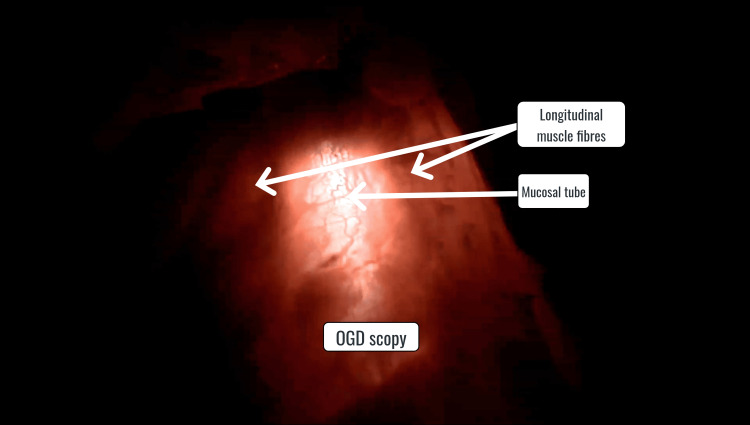
Laparoscopic view of mucosal tube on OGD scopy OGD: oesophagogastroduodenoscopy

These bands of muscle fibers were subsequently divided to complete the myotomy. Except for one patient, no intraoperative leak of ICG was observed, thereby confirming the absence of perforation. The completeness of myotomy and the absence of mucosal perforation were further confirmed by OGD scopy (Figure 4).

An underwater leak test was performed, and no air bubbles were visualized. Dor’s fundoplication was performed in all the patients. No drains were placed.

## Results

**Figure 5:**
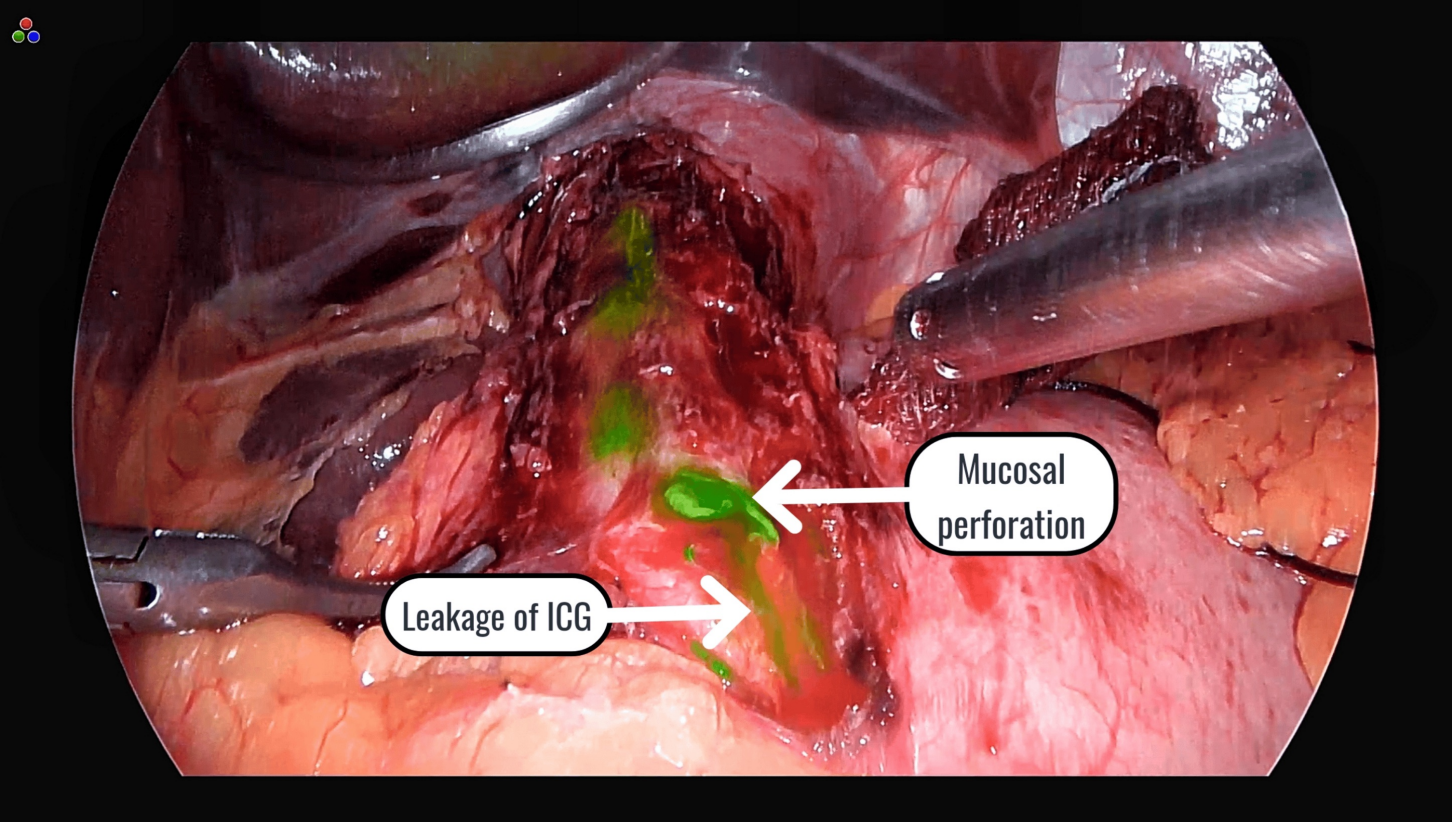
Mucosal perforation identified by leakage of ICG ICG: indocyanine green

In this study, Heller's cardiomyotomy with Dor's fundoplication was performed on 15 patients, and ICG was used intraoperatively to assess the completeness of the myotomy and the integrity of the mucosa. Among the 15 patients, one individual who had previously undergone esophageal dilatation experienced an inadvertent mucosal perforation, which was identified due to intraoperative leakage of ICG (Figure 5).

The mucosal perforation was closed using polyglactin 3-0, and the postoperative period was uneventful. A CT scan of the abdomen with oral contrast was performed on postoperative day 3, which showed no leakage of the orally administered contrast. Consequently, the nasogastric tube was removed, and the patient was discharged on postoperative day 4 on a liquid diet.

For the remaining patients, no intraoperative leakage of ICG was observed, which was further confirmed through intraoperative OGD scopy and an underwater leak test. The nasogastric tube was removed at the end of the surgery. All patients were maintained on a liquid diet postoperatively for one week and were discharged on postoperative day 2 without complications.

At the first follow-up visit, conducted one week after the surgery, all patients were asymptomatic, and they were transitioned to a semisolid diet. During the six-month follow-up, patients continued to be asymptomatic, and no further radiological investigations were necessary.

The patient outcomes of our study are mentioned in Table 1.

**Table 1:** Patient outcomes

Patient no	Age (in years)	Sex	Surgery time (in mins)	Was there leakage of ICG	Approximate amount of blood loss (in ml)	Number of days until discharge	Symptoms on 6 monthly follow up
1	34	Male	66	No	50	2	Asymptomatic
2	39	Male	58	No	60	2	Asymptomatic
3	41	Female	71	No	50	2	Asymptomatic
4	28	Male	62	No	40	2	Asymptomatic
5	54	Female	67	No	50	2	Asymptomatic
6	26	Female	56	No	50	2	Asymptomatic
7	33	Female	64	No	40	2	Asymptomatic
8	31	Male	63	No	50	2	Asymptomatic
9	48	Female	76	No	60	2	Asymptomatic
10	27	Male	98	Yes	90	4	Asymptomatic
11	24	Male	57	No	40	2	Asymptomatic
12	45	Male	65	No	40	2	Asymptomatic
13	34	Female	70	No	50	2	Asymptomatic
14	52	Female	68	No	50	2	Asymptomatic
15	37	Male	57	No	50	2	Asymptomatic

## Discussion

Intraoperative endoscopy is recommended by many experts because it may be useful in guiding the completeness of the myotomy, preventing esophageal narrowing, and detecting leaks intraoperatively [7]. However, logistical issues and the non-availability of intraoperative endoscopy may limit the utility of intraoperative endoscopy.

ICG has been utilized in laparoscopic and robotic surgeries to facilitate the visualization for a detailed understanding of the biliary anatomy. It is an amphiphilic, tricarbocyanine iodide dye (mass = 751.4 Da) that is reconstituted in an aqueous solution of pH 6.5 for intravenous injection in patients [8-10].

ICG offers high contrast and sensitivity due to the use of near-infrared light to measure fluorescence, which makes tissues appear more translucent [8]. The ICG molecule has an excitation wavelength ranging between 750 nm and 800 nm, and fluorescence is viewed around the maximum peak of 832 nm [11,12].

ICG dye has been widely utilized in modern times for bioimaging in surgeries to facilitate the identification of obscure and frequently difficult-to-find anatomical structures [13]. Through adequate visualization of tissues at depth and those within the dissection area, near-infrared imaging helps to avert surgical disasters that could arise from unintentional damage to surrounding vital structures [13]. In our previous studies, we have shown the value of ICG in laparoscopic cholecystectomy [14], laparoscopic completion cholecystectomy for stump cholecystitis [15], and visualization of ureters in complex laparoscopic pelvic surgeries [16]. ICG is also useful in checking the vascularity of the stomach in cases of redo fundoplications, where the stomach has volvulated.

The lack of standardization regarding adequate dosage of ICG for the best possible intraoperative visualization of the mucosal tube requires further evaluation. On comparing the dose of 4 mg in 100 ml NS in our pilot study [6] to a dose of 10 mg in 100 ml NS, we found that 10 mg in 100 ml NS gave better intraoperative visualization in terms of higher contrast, and therefore, we standardized this dosing in the current study.

ICG is a practical tool for evaluating the completeness of cardiomyotomy and identifying any intraoperative mucosal perforations. To use this tool, you only need an ICG-enabled vision system and a nasogastric tube, which could be easily handled by either the anesthetist or a surgical assistant. It is particularly beneficial in settings where intraoperative endoscopy or a trained endoscopist is not available.

In our more than 10 years of experience performing over 200 laparoscopic Heller’s cardiomyotomies, we propose that ICG can become a useful tool for confirming the completeness of myotomy. After our previous study about the feasibility of redo laparoscopic Heller’s cardiomyotomy [17], we suggest that ICG may become a useful tool where myotomy is difficult due to fibrosis.

Limitations

Although the results of using ICG in achalasia cardia are promising, the sample size in this study is small, and further studies, including a larger sample size and a control group where ICG is not used, could help assess the efficacy of this technique.

## Conclusions

The use of near-infrared fluorescence imaging with ICG dye offers an effective alternative to intraoperative OGD scopy for confirming the completeness of myotomy and ensuring an accurate assessment of the myotomy site. It provides a safe and reliable method to verify the procedure's success, especially in situations where intraoperative OGD scopy may not be available or feasible. Additionally, this approach may offer cost-saving benefits, potentially reducing the need for additional equipment or specialized personnel, making it a practical option in resource-limited settings.
